# Synthesis, Characterization and Biological Evaluation of Co(II), Cu(II), Ni(II) and Zn(II) Complexes With Cephradine

**DOI:** 10.1155/MBD.2000.265

**Published:** 2000

**Authors:** Zahid H. Chohan, Maimoon F. Jaffery

**Affiliations:** Department of Chemistry Islamia University Bahawalpur Pakistan

## Abstract

Some Co(II), Cu(II), Ni(II) and Zn(II) complexes of antibacterial drug cephradine have been prepared and characterized by their physical, spectral and analytical data. Cephradine acts as bidentate and the complexes have compositions, [M(L)_2_X_2_] where [M = Co(II), Ni(II) and Zn(II), L = cephradine and X = Cl_2_] showing octahedral geometry, and [M(L)_2_] where [M = Cu(II), L = cephradine] showing square planar geometry. In order to evaluate the effect of metal ions upon chelation, eephradine and its complexes have been screened for their antibacterial activity against bacterial strains, *Escherichia coli*, *Staphylococcus aureus*, and *Pseudomonas aeruginosa*.


### SYNTHESIS,CHARACTERIZATION AND BIOLOGICAL EVALUATION OF Co(II), Cu(II), Ni(II) AND Zn(II) COMPLEXES WITH CEPHRADINE

Zahid H. Chohan* and Maimoon F. Jaffery

Department of Chemistry, Islamia University, Bahawalpur, Pakistan

### ABSTRACT
Some Co(II), Cu(II), Ni(II) and Zn(II) complexes of antibacterial drug cephradine have been prepared and characterized by their physical, spectral and analytical data. Cephradine acts as bidentate and the complexes have compositions, $[M(L)_2X_2]$ where [M=Co(II), Ni(II) and Zn(II), L=cephradine and $X=Cl_2$] showing octahedral geometry, and $[M(L)_2]$ where [M=Cu(II), L=cephradine] showing square planar geometry. In order to evaluate the effect of metal ions upon chelation, cephradine and its complexes have been screened for their antibacterial activity against bacterial strains, *Escherichia coli*, *Staphylococcus aureus*, and *Pseudomonas aeruginosa*

### INTRODUCTION
Evidences supporting the introduction of metallic elements in several biological processes are rapidly accumulating[1-6]. Schubert[7] and Kirschner[8] have investigated the antibacterial, antiviral and anticancer activities of more than 25 inorganic compounds, which included the metal atom as potentially significant part of the molecule. They suggested[8,9] that the transfer of metal ion from the ligand to the cancer-associated viruses was a mechanism for releasing the anticancer drug in the locality of the tumor. Due to the significant nature of the metallic ions, their metal complexes are now, being included in the search for ideal anticancer drugs. Such significant examples of which are, palladium and platinum complexes of 6-mercaptopurine that destroy[10] adenocarcinomas and similarly, the complexes of dialkyldithiophosphate which reduce some tumors[11]. A wide range of activities of even the simplest complexes of metallic elements has been reviewed by Rosenberg[12] who noticed that the viral-induced cancers all respond to the treatment with even the simplest metal-amine halide complexes. It has also been demonstrated that chelation/complexation tend to make inactive substances/ligands active and active compounds/drugs become more active and less toxic[13-16]. All these evidences however, highlight the need to study and evaluate more the biological applications of metallic elements for therapeutic potentials.

Cephradine is a first-generation cephalosporin class of antibiotic[17], actively used against Gram-positive cocci, and Gram-negative bacilli. It has similar structural and antibacterial relationship to that of its closely related analogue cephalexin. It contains the $(NH_2)$, (COOH), (NH) and (C=O) functional groups and its molecular model reveals that its structure is suitable for chelation/complexation. A detailed biological evaluation of the copper(II) and Zn(II) complexes of cephalexin has been reported[18]. In order to evaluate the biological comparison of both the analogues, we, therefore, report in this paper the preparation, characterization and biological properties of cobalt(II), copper(II), nickel(II) and Zn(II) complexes of cephradine which may be an addition to reveal the significance of the strong relationship between metals, or their complexes, and bactericidal activity.

### EXPERIMENTAL
### Material and Methods
All chemicals and solvents used were of Analar grade. All metal(II) salts were used as chlorides. Sodium salt of cephradine was obtained by reacting equimolar amount of sodium hydroxide with cephradine. IR spectra were recorded on a Philips Analytical PU 9800 FTIR spectrophotometer as KBr discs. UV-Visible spectra were obtained in DMF on a Hitachi U-2000 double-beam spectrophotometer. C, H and N analyses was carried out by Butterworth Laboratories Ltd. Conductance of the metal complexes was determined in DMF on a Hitachi YSI-32 model conductometer. Magnetic measurements were made on solid complexes using the Gouy method. Melting points were recorded on a Gallenkamp apparatus and are uncorrected.
### Preparation of cobalt(II)-cephradine Complex
An ethanol solution (20 mL) of the cobalt chloride hexahydrate (0.24 g, 0.001 M) was added to a magnetically stirred sodium salt of cephradine (0.8 g, 0.002 M) in distilled water (25 mL). The mixture was refluxed for 1 h and cooled to room temperature. On cooling, pink precipitates were formed, filtered and washed with ethanol, acetone and ether, and dried by suction. Crystallization in aqueous ethanol (70 : 30) gave the desired metal complex. All other metal complexes were formed following the same method.
### Antibacterial Studies
The synthesized metal complexes, in comparison to the uncomplexed cephradine were screened for their antibacterial activity against pathogenic bacterial species, *Escherichia coli*, *Staphylococcus aureus* and

*Pseudomonas aeruginosa*. The paper disc diffusion method was adopted for the determination of antibacterial activity[14-16].

### RESULTS AND DISCUSSION

All the complexes are intensely colored and stable amorphous solids, which did not have sharp melting points and decompose above 225°C. The complexes are only soluble in DMF and DMSO and insoluble in all other common organic solvents. Their melting behavior, solubility and crystalline nature suggest that they are monomers. Molar conductance values (14-18 $ohm^{-1}cm^{2}mol^{-1}$) of the complexes in DMF solution indicated[19] that all of the complexes are non-electrolytes.

Table 1. Physical and Analytical Data of the Metal(II) Complexes

| Complex/ Mol. Formula | M.p (°C) | B.M. ($\mu_{eff}$) | Yield (%) | Calc. (Found) % C H N Cl |
|---|---|---|---|---|
| Cephradine | 218 | - | - | - |
| (1) $[Co(L)_2(Cl)_2]$ $C_{32}H_{36}CoCl_2N_6O_8S_2$ [956.9] | 232-234 | 4.7 | 60 | 40.1 3.8 8.8 7.4 (39.9)(3.5)(8.9)(7.2) |
| $[Cu(L)_2]$ $C_{32}H_{36}CuN_6O_8S_2$ [890.7] | 228-230 | 1.4 | 62 | 43.1 4.0 9.4 - (43.4)(3.9)(9.7) |
| $[Ni(L)_2(Cl)_2]$ $C_{32}H_{36}NiCl_2N_6O_8S_2$ [956.7] | 231-233 | 3.3 | 58 | 40.1 3.8 8.8 7.4 (40.5)(4.1)(8.6)(7.3) |
| $[Zn(L)_2(Cl)_2]$ $C_{32}H_{36}ZnCl_2N_6O_8S_2$ [963.4] | 234-236 | Dia | 62 | 39.8 3.7 8.7 7.3 (40.1)(3.5)(8.9)(7.5) |

Examination of the physical model of cephradine shows that it can exhibit either ONON, OONO or OOON donor quadridentate, ONO, OOO, NNO or NNN donor tridentate and NO, OO, or NN donor bidentate behavior. The molecular model studies further reveal that in no cases the ligand can stereochemically behave as quadridentate or tridentate.

The IR spectra of the complexes in comparison to the uncomplexed cephradine are listed in Table 2 with some tentative important characteristic assignments[20]. The IR spectrum of the cephradine shows some characteristic bands at 3345, 3240, 1770 and 1745 mainly due to the $\nu(NH_2)$, $\nu(NH)$, $\nu(COOH)$ and $\nu(C{=}O)$ vibrations, respectively. The metal complexes contained all the bands from cephradine and also other bands indicative of the coordination of the ligand with the metal ions. The band due to $\nu(COO)_{asym}$ at 1770 $cm^{-1}$ in the spectrum of the ligand shifted to lower frequency (10-15 $cm^{-1}$) in all the metal complexes indicative of the complexation[21]. A new absorption band instead, assigned to $\nu(COO)$ and $\nu(M\text{-}O)$ appeared at 1565-1575 and 450-455 $cm^{-1}$ which were only observed in the spectra of the complexes. This in turn, indicated that the carboxyl group is coordinated to the metal ion. Also, the band due to $\nu(NH_2)$ at 3345 $cm^{-1}$ was found shifted to lower wave number (15-20 $cm^{-1}$) in the spectra of its metal complexes. And a new band at 415-420 $cm^{-1}$ assigned to $\nu(M\text{-}N)$ was evolved in the spectra of the complexes indicating[22] involvement of the $\nu(NH_2)$ group via nitrogen in the coordination. Moreover, In the far infrared region a new band at 335 $cm^{-1}$ was found in the spectra of the Co(II), Ni(II) and Zn(II) complexes and not observed in the spectrum of the Cu(II) complex assigned to $\nu(M\text{-}Cl)$ modes respectively. It in turn, indicated that two chloride atoms are also coordinated to the metal atom. These evidences however, indicated that the Co(II) Ni(II) and Zn(II) complexes possess an octahedral geometry (Fig 1A) and Cu(II) complex to show square planar geometry (Fig.1B).

$^{1}H$ and $^{13}C$ NMR spectra of the uncomplexed cephradine and its metal complexes have been recorded in DMSO-$d_6$ with TMS as internal reference and are summerized in Table 2 with some tentative assignments. The spectra of cephradine exhibited peaks as expected[23]. Signals for the NH and COOH protons in the spectra of uncomplexed Cephradine at 11.1 and 10.8 ppm disappeared in the complexes. This indicated that Cephradine is coordinated to the metal ions by deprotonation. In the complexes the aromatic proton signals appeared downfield due to increased conjugation during coordination[24,25].

UV-Visible spectral bands of the complexes are recorded in Table 2. The cobalt(II) complex shows magnetic moment 4.7 B.M. at room temperature (Table. 1). These high value of the magnetic moment and the stoichiometry suggest a coordination number of six for the central cobalt(II) ion and an octahedral geometry. The electronic spectrum of the complex is consistent with its octahedral environment around the cobalt(II) ion (Fig.1A). The spectrum displays two bands at 20,715 and 28,580 $cm^{-1}$ attributed to $^{4}T_{1g} \rightarrow {}^{4}T_{1g}$ and $^{4}T_{1g} \rightarrow {}^{4}T_{2g}$ transitions, respectively, in a high-spin octahedral geometry[26,27].

Table 2. IR, UV and NMR Spectroscopic Data of the Metal(II) Complexes

| Cephradine/ Complex | IR ($cm^{-1}$) | $\lambda_{max}$ ($cm^{-1}$) | $^1H$-NMR (DMSO-$d_6$) (ppm) | $^{13}C$-NMR (DMSO-$d_6$) (ppm) |
|---|---|---|---|---|
| Cephradine | 3345 (s, $NH_2$), 3240 (ms, NH), 1770 (s, COOH), 1745 (s, C=O) | - | 2.3 (s, 6H, $CH_3$), 4.5 (d, 2H, NCH), 7.1 (m, 4H, Ph), 7.2 (m, 4H, Ph), 7.4 (m, 2H, Ph), 8.1 (d, 2H, β-Lactam), 8.3 (m, 4H, hetero-Ph), 8.4 (m, 2H, hetero-Ph). | 13 ($CH_3$), 63 (CHN), 117 (β-Lactam), 120, 122, 124 (hetero-Ph), 148, 125, 127, 128, 172 (C=O), 177 (COO), 212 (β-Lactam). |
| 1 | 3530 (s, $NH_2$),1760, 1565 (s, COO), 450 (m, M-N), 420 (m, M-O), 355 (m, M-Cl) | 20715, 2850 | 2.3 (s, 6H, $CH_3$), 4.5 (d, 2H, NCH), 7.1 (m, 4H, Ph), 7.2 (m, 4H, Ph), 7.4 (m, 2H, Ph), 8.1 (d, 2H, β-Lactam), 8.3 (m, 4H, hetero-Ph), 8.4 (m, 2H, hetero-Ph). | 13 ($CH_3$), 63 (CHN), 117 (β-Lactam), 120, 122, 124 (hetero-Ph), 148, 125, 127, 128, 172 (C=O), 177 (COO), 212 (β-Lactam). |
| 2 | 3535 (s, $NH_2$),1755, 1565 (s, COO), 455 (m, M-N), 425 (m, M-O) | 15155, 18575, 30245 | 2.3 (s, 6H, $CH_3$), 4.5 (d, 2H, NCH), 7.1 (m, 4H, Ph), 7.2 (m, 4H, Ph), 7.4 (m, 2H, Ph), 8.1 (d, 2H, β-Lactam), 8.3 (m, 4H, hetero-Ph), 8.4 (m, 2H, hetero-Ph). | 13 ($CH_3$), 63 (CHN), 117 (β-Lactam), 120, 122, 124 (hetero-Ph), 148, 125, 127, 128, 172 (C=O), 177 (COO), 212 (β-Lactam). |
| 3 | 3535 (s, $NH_2$),1762, 1570 (s, COO), 455 (m, M-N), 425 (m, M-O), 355 (m, M-Cl) | 16570, 27375 | 2.3 (s, 6H, $CH_3$), 4.5 (d, 2H, NCH), 7.1 (m, 4H, Ph), 7.2 (m, 4H, Ph), 7.4 (m, 2H, Ph), 8.1 (d, 2H, β-Lactam), 8.3 (m, 4H, hetero-Ph), 8.4 (m, 2H, hetero-Ph). | 13 ($CH_3$), 63 (CHN), 117 (β-Lactam), 120, 122, 124 (hetero-Ph), 148, 125, 127, 128, 172 (C=O), 177 (COO), 212 (β-Lactam). |
| 4 | 3533 (s, $NH_2$),1760, 1565 (s, COO), 450 (m, M-N), 422 (m, M-O), 355 (m, M-Cl) | 27555 | 2.3 (s, 6H, $CH_3$), 4.5 (d, 2H, NCH), 7.1 (m, 4H, Ph), 7.2 (m, 4H, Ph), 7.4 (m, 2H, Ph), 8.1 (d, 2H, β-Lactam), 8.3 (m, 4H, hetero-Ph), 8.4 (m, 2H, hetero-Ph). | 13 ($CH_3$), 63 (CHN), 117 (β-Lactam), 120, 122, 124 (hetero-Ph), 148, 125, 127, 128, 172 (C=O), 177 (COO), 212 (β-Lactam). |

s=sharp, ms=medium sharp, m=medium

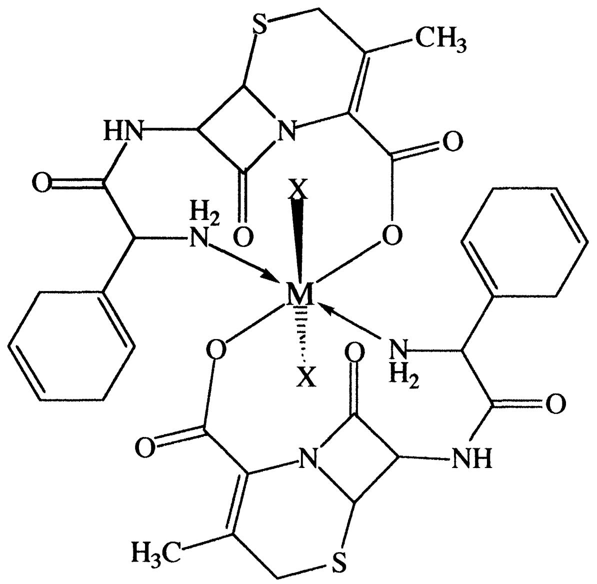

[1A] M=Co(II), Ni(II) or Zn(II); X=Cl

[1B] M=Cu(II); X=0

Fig. 1. Proposed Structure for the Metal(II) Complexes

The Cu(II) complex shows two weak low-energy bands at 15,155 $cm^{-1}$ and 18575 $cm^{-1}$ and a strong high-energy band at 30,245 $cm^{-1}$. The low-energy bands are in positions typically found for square planar configuration and may be assigned to ${}^2B_{1g} \rightarrow {}^2A_{1g}$ and ${}^2B_{1g} \rightarrow {}^2E_g$ transitions respectively[28,29]. The strong high-energy band is assigned to metal → ligand charge transfer. Also, the magnetic moment value (1.4 B.M) for the Cu(II) complex is found to be consistent with the proposed square planar geometry (Fig 1B) for the Cu(II) complex.

The electronic spectra of the nickel(II) complex exhibited absorption bands at 16,570 and 27,375 $cm^{-1}$, attributed to ${}^3A_{2g} \rightarrow {}^3T_{1g}$ (F) and ${}^3A_{2g} \rightarrow {}^3T_{1g}$ (P) transitions, respectively, in an octahedral geometry[30,31]. The calculated values of the ligand field parameter lie in the range reported for an octahedral structure. Also, the value of the magnetic moment (3.3 B.M.) may be taken as an additional evidence for its octahedral structure.

The diamagnetic zinc(II) complex did not show any d-d bands and its spectrum is dominated only by the charge transfer band. This charge transfer band at 27,555 $cm^{-1}$ was assigned to the transition ${}^2E_g \rightarrow {}^2T_{2g}$, possibly in an octahedral environment[32].

On the basis of the above observations, it is tentatively suggested that the Co(II), Ni(II) and Zn(II) complexes show an octahedral geometry (Fig. 1A) in which the two cephradine molecules act as bidentate and at axial positions two chlorides are coordinated to the metal atom. In the Cu(II) complex the two cephradine molecules respectively, acting as bidentate show a square planar geometry of the complex (Fig. 1B) by possibly accommodating themselves around the metal atom in such a way that a stable chelate ring is formed.

**Antibacterial Properties**

Cephradine in comparison to its metal(II) chelates was evaluated for antibacterial activity against the standard bacterial strains, *Escherichia coli (a),), Staphylococcus aureus (b)* and *Pseudomonas aeruginosa (c)* The compounds were tested at a concentration of 30 μg/0.01 mL in DMF solution using the paper disc diffusion method reported[33,34] as earlier. The susceptibility zones were measured in diameter (mm) and the results are reproduced in Table 3. The susceptibility zones were the clear zones around the discs. Cephradine and its complexes individually were found to be biologically active showing various degrees of inhibitory effects on the growth of the tested bacterial species. The antibacterial results evidently show that the complexation improved the antibacterial activity. These observations however, suggest that the metallic elements should be seriously considered in <drug designing>. Their use could provide an easy way of improving the bactericidal activity of many drugs/antibacterial agents.

Table 3. Antibacterial Activity Data of Cephradine and its Metal(II) Complexes

| Cephradine/ Complex | Microbial Species | | |
|---|---|---|---|
| | a | b | c |
| Cephradine | ++ | + | ++ |
| (1) | +++ | ++ | +++ |
| (2) | ++++ | +++ | +++ |
| (3) | +++ | ++ | ++++ |
| (4) | +++ | +++ | ++ |

a= *Escherichia coli*, b= *Staphylococcus aureus*,
c= *Pseudomonas aeruginosa.* Inhibition zone diameter mm (% inhibition): +, 6-10 (27-45 %); ++, 10-14 (45-64 %); +++, 14-18 (64-82 %); ++++, 18-22 (82-100 %). Percent inhibition values are relative to inhibition zone (22 mm) with 100 % inhibition.

### ACKNOWLEDGEMENT

The authors gratefully acknowledge the help of the Department of Microbiology, Qaid-e-Azam Medical College, Bahawalpur, in performing the antibacterial studies.

### REFERENCES

1. H. Sigel and D. B. McCormick, *Accts. Chem. Res.*, **3**, 201 (1970).
2. M. Dixon and E. C. Webb, "*Enzymes*", Green and Co, London, (1964).
3. E. G. Sanders, L. D. Wright and D. B. McCormick, *J. Biol. Chem*, **240**, 3628 (1965).
4. D. R. Williams, "*The Metals of Life*", Van Nostrand, London, (1971).
5. M. J. Seven and L. A. Johnson, "*Metal Binding in Medicine*", Lippincott Co, Philedelphia, (1960).
6. F. P. Dwyer and D. P. Mellor, "*Chelating agents and Metal Chelates*", Academic Press, London, (1964).
7. J. Schubert, *Sci. Amer*, **214**, 40 (1966).
8. S. Kirschner, Y. K. Wei, D. Francis and D. Bergman, *J. Med. Chem*, **9**, 369 (1966).
9. S. Kirschner, S. H. Kravitz and J. Mack, *J. Chem. Documentation*, **6**, 213 (1966).
10. S. E. Livingstone and J. D. Nolan, *Inorg. Chem*, **7**, 1447 (1968).
11. S. E. Livingstone and J. D. Nolan and A. E. Mihkelson, *Inorg. Chem*, **9**, 2545 (1970).
12. B. Rosenberg, *Plat. Met. Rev*, **15**, 42 (1971).
13. J. R. J. Sorenson, *J. Appli. Nutr*, **32**, 4 (1980).
14. J. R. J. Sorenson, "*Inflammatory Diseases and Copper*", Human Press, NJ, (1982).
15. R. Nagar and G. Mohan, *J. Inorg. Biochem*, **42**, 9 (1991).
16. S. Oga, S. F. Taniguchi, R. Najjar and A. R. Souza, *J. Inorg. Biochem*, **41**, 45 (1991).

17. H. R. Mahler and E. H. Cordes, "Biological Chemistry", Harper and Rowe, New York, (1966).
18. M. S. Iqbal, A. R. Ahmad, M. Sabir and S. M. Asad, *J. Pharm. Pharmacol*, **51**, 371 (1999).
19. W. J. Geary, *Coord. Chem. Rev*, **7**, 81 (1971).
20. L. J. Bellamy, "*The Infrared Spectra of Complex Molecules*", 3rd Ed, Methuen, London, (1966).
21. M. Yongxiang, Z. Zhengzhi, M. Yun and Z. Gang, *Inorg. Chim. Acta*, **165**, 185 (1989).
22. K. Nakamoto, "*Infrared Spectra of Inorganic and Coordination Compounds*", 2nd Ed, Wiley Interscience, New York, (1970).
23. G. Dunn, J. Antibiot, **29**, 65 (1976).
24. Z. Gang, L. Feng, X. Jishan and M. Yongxiang, Polyhedron, **7**, 303 (1988).
25. Z. Hong-Yum, C. Dong-Li, C. Pei-Kun, C. De-Ji, C. Guang-Xia and Z. Hong-Quan, Polyhedron, **11**, 2313 (1992).
26. B. N. Figgis, "*Introduction to Ligand Fields*", J. Wiley, New York, (1976).
27. B. P. Lever, "*Inorganic Electronic Spectroscopy*", Elsevier, Amsterdam, (1984).
28. D. Liehr, *J. Phys. Chem*, **67**, 1314 (1967).
29. R. L. Carlin,., "*Transition Metal Chemistry*", Ed, R. L. Carlin, Vol 1, Marcel Decker, New York, (1965).
30. D. W. Meek, R. S. Drago and T. S. Piper, *Inorg. Chem.*, **1**, 285 (1962).
31. R. S..Drago, "Physical Methods in Inorganic Chemistry", Reinhold, New York, (1965).
32. B. N. Figgis and J. Lewis, *Prog. Inorg. Chem.*, **6**, 87 (1964).
33. Z. H. Chohan and S. K. A. Sherazi, *Synth. React. Inorg. Met.-Org. Chem*, **29**, 105 (1999).
34. Z. H. Chohan, M. Praveen and A. Ghaffar, *Synth. React. Inorg. Met.-Org. Chem*, **28**, 1673 (1998).

**Received: September 20, 2000 - Accepted: November 2, 2000 - Received in revised camera-ready format: January 16, 2001**

